# The Response of the Indonesian Dental Community to the COVID-19 Pandemic

**DOI:** 10.1016/j.identj.2024.01.004

**Published:** 2024-02-16

**Authors:** Iwan Dewanto, Rosa Amalia, Armelia Sari Widyarman, Freddy W Ferdiansyah

**Affiliations:** a Department of Dental Public Health, Faculty of Dentistry, Universitas Muhammadiyah Yogyakarta, Indonesia; b Department of Preventive and Community Dentistry, Faculty of Dentistry, Universitas Gadjah Mada, Yogyakarta, Indonesia; c Department of Microbiology, Faculty of Dentistry, Universitas Trisakti, Jakarta, Indonesia; d Indonesia Dental Association, East Jakarta, DKI Jakarta, Indonesia

**Keywords:** COVID-19, Knowledge, Awareness, Response, Infection control

## Abstract

**Objective:**

The aim of this study was to assess the response of dental health care workers in Indonesia to the COVID-19 pandemic through (1) a countrywide web-based, questionnaire survey of their knowledge, attitude, and infection control (IC) practices during the pandemic and (2) a focus group discussion (FGD) on the latter aspects with infected personnel, as well as (3) archival data collection on the epidemiology of COVID-19 amongst dentists in Indonesia.

**Methods:**

A questionnaire survey using a web platform was conducted in May 2020 using a simple random sample of 3586 dentists working in different regions of Indonesia. The questionnaire attempted to elicit their knowledge and attitudes towards COVID-19, implementing IC measures, and providing dental care during the outbreak. The questionnaire comprised sections assessing participants' knowledge, awareness, and IC practice. In this study, the data were subjected to analysis through descriptive statistics and one-way analysis of variance (ANOVA) with a predetermined significance level of <.05. The study's second phase involved an FGD with dentists who had contracted the disease to discuss the impediments they faced during dental practice. We collected data on the number of dentists with COVID-19 from May 2020 to March 2022.

**Results:**

The questionnaire survey revealed that the dentists had a satisfactory understanding of COVID-19 spread and the IC measures required to curb disease spread in the dental clinic. Of note, some dentists lacked knowledge of the incubation period of 5 days (21.19%). The vast majority of the respondents (>80%) were aware of the nature of the SARS-CoV-2 virus. Community health centres and the personnel who served there were the most exposed health care providers.

**Conclusions:**

Results suggest that the vast majority of the dental care professionals in Indonesia had adequate knowledge and awareness of COVID-19. However, some aspects of practice need to be improved in IC and related aspects.

## Introduction

The SARS-CoV-2 virus caught the world off-guard in early 2020. It spread globally within a relatively short period, with the World Health Organization (WHO) declaring it a pandemic and a public health emergency of international concern in January 2020. COVID-19 is caused by the novel coronavirus SARS-CoV-2, first reported in December 2019 in the Chinese province of Wuhan. Many jurisdictions took COVID-19 prevention and control measures in the wake of the pandemic. The WHO also outlined the detailed global surveillance and the pandemic preparedness plan for COVID-19 simultaneously.^1^, ^2^, ^3^, ^4^

The main route of COVID-19 human-to-human transmission is through respiratory droplets, particularly during coughing and sneezing, as well as from aerosols. The disease is also transmitted through contact with an infected person or through fomites and other contaminated surfaces.^5^ In general, the incubation period of COVID-19 is 2 to 14 days. The signs and symptoms of COVID-19 are highly variable, ranging from asymptomatic infection to life-threatening disease depending on the comorbidities of infected individuals.^5^ A significant proportion of the infected, approximately 40%, can be asymptomatic, leading to diagnostic difficulties and challenges in mitigating the spread of the disease.^6^, ^7^, ^8^

In the vast majority of individuals, COVID-19 infection can be prevented by successful vaccination, and personal protection, face masks, regular handwashing, and social distancing best prevent community spread of COVID-19.^9^ Health care professionals (HCPs), particularly dental professionals, were considered a high-risk population, as dental surgical procedures create a high volume of contaminated aerosols in the close vicinity of clinical personnel.^10^, ^11^, ^12^

In Indonesia, there have been 3 categories of COVID-19 waves: the first phase (COVID-19 type Alpha) from May through October 2020, the second phase (COVID-19 type Delta) from November 2020 through August 2021, and the third phase (COVID-19 type Omicron) from November 2021 through March 2022. The first case of COVID-19 in Indonesia was reported on March 2, 2020. A month later, the Indonesian task force for the COVID-19 pandemic (SATGAS) announced that dental professionals should be considered a high-risk group and urged dentists to abate dental services temporarily. A few months later, in August 2020, Indonesia reported more than 121,000 confirmed cases of the disease (https://covid19.go.id/peta-sebaran).

There was a simultaneous effort by the Indonesian Dental Association (IDA) to mitigate infection risk. The IDA published a handbook on guidelines and strict protocols that must be followed for dental practice during the pandemic period. The IDA also sent a missive to all Indonesian dentists to restrict services only to emergency patients, as by this time, 8 dentists had succumbed to COVID-19, possibly contracted through patient care, and also due to the dearth of personal protective equipment (PPE) in Indonesia. In a subsequent letter, the IDA emphasised the importance of using PPE level 3 when handling all patients, irrespective of their health status.

As dentists are key health professionals, it is pivotal that they are armed with the proper knowledge and attitudes towards this infection.^13^, ^14^, ^15^, ^16^ Further, as mentioned, during the primary and subsequent waves of the pandemic, many guidelines were issued to Indonesian dentists by IDA and other health organisations. Nonetheless, dentists' awareness, knowledge, attitude, and behaviour related to the disease and adherence to infection control (IC) measures are yet unknown.^10^^,^^17^^,^^18^ Hence, the primary aim of this study was to assess the knowledge, awareness, and attitudes of dental health care workers to the COVID-19 pandemic through a countrywide survey of the Indonesian archipelago. Our core hypothesis for the study therefore was that the knowledge, attitudes, and IC measures adopted by dentists in Indonesia were adequate throughout the pandemic period.

A secondary objective was forming a focus group study of 54 dentists who contracted COVID-19 to discuss mainly the impediments they faced in delivering safe dental care during the pandemic. Last, we attempted to ascertain the total number of dentists who contracted the disease in the first, second, and third waves of the disease spread through records available from the COVID-19 task force in Indonesia and the IDA.

## Materials and methods

The study comprised 3 major components. The first was a cross-sectional questionnaire survey of 3586 dentists conducted from May through July 2020 to assess their knowledge and attitudes on COVID-19, how they implemented IC measures, and the provision of care during the outbreak. Participation in the study was voluntary, and identification information was not collected from the study participants. An online questionnaire using Google Forms was used to collect the data.

The second part of the study comprised focus group discussions (FGDs) that were also held twice during the second wave (August 14, 2021) and the third wave of COVID-19 (January 10, 2022) with the dentists who contracted the disease. The lead author (ID) conducted the FGDs using the Zoom platform. Finally, we collected IDA reports for 2 years, from May 2020 through March 2022, on the number of dentists exposed to COVID-19 and those who succumbed, contracting the disease either through clinical practice or otherwise. The data were obtained from all 270 branches of the IDA, each of which had a COVID-19 task force.

Ethical clearance for all study phases was obtained from the Ethics Committee of the Faculty of Dentistry Universitas Trisakti Jakarta (No. 346/S3/KEPK/FKG/5/2020). In addition, informed consent was obtained from the participants confirming their willingness to participate in the study.

### Questionnaire design

A structured multiple-choice questionnaire that was pretested with 3 major components was used: first, the sociodemographic characteristics of the cohort; second, their knowledge of COVID-19 and the disease spread and containment; and finally, the dental practice and modifications during the COVID-19 pandemic, including patient management and IC aspects.

For the knowledge component on COVID-19, we queried their knowledge of the incubation period and the signs and symptoms of the disease, the mode of transmission, and disease spread. Also questioned were their patient management protocols and the implementation of IC procedures, with particular emphasis on airborne and droplet precautions, as dictated by the IDA and US Centers for Disease Control and Prevention (CDC).^19^ Analysis of variance (ANOVA) was performed to detect differences in knowledge and practice components amongst all groups using SPSS software. A significance level <.05 was considered significant.

### FGDs

The objective of the FGDs, conducted under the auspices of the IDA, was to discover the profile of dentists who contracted COVID-19 in their workplace and the weak links in disease transmission. It was assumed that the disease was transmitted in the clinic, as they actively practiced during the pandemic.

Four experts developed the guiding questions for the FGDs in consultation with the members of the COVID-19 task force and representatives of the IDA. Five questions guided the FGD, and the moderators asked questions from the dentists as per the discussion guide to evaluate how the dentists may have contracted COVID-19 and the issues they faced in executing safe dental practice during the pandemic.

All recorded COVID-19–infected dentists were invited for the FGD remotely conducted via Zoom, and a representative sample of 54 participated. Most (85%; n = 46) worked in community health centres, whilst the remainder were in hospital care services. Participation in the FGD was voluntary, and personal identification information was not collected from the study cohort.

## Results

The Google Forms questionnaire was completed by a total of 3586 dentists from diverse regions across Indonesia, most of whom were female (78.7%). The response rate of the questionnaire was 23.9%. Almost a third were in community dental services, whilst one-quarter were in hospital practice (Table 1).

**Table 1:** Demographic characteristics of dentists enrolled in the questionnaire study (N = 3586).

Profile	No.	%
Gender		
Female	2823	78.73
Male	763	21.27
Age, y		
20–30	781	21.78
31–40	1321	36.83
40–50	820	22.87
>50	664	18.52
Place of work		
Lecturer/researcher	181	5.05
Community health centre	1162	32.40
Private dental clinics	673	18.77
Primary care clinics	556	15.50
Private hospital	366	10.21
Public hospital	497	13.86
Secondary care clinic	123	3.43
Other	28	0.78
Practice experience		
1-10 years	1758	49.02
11-20 years	936	26.10
>20 years	801	22.34
No answer	91	2.54

The vast majority of the respondents (>80%) were aware of the nature of SARS-CoV-2, the disease's spread routes, and the increased severity of the disease in those with comorbidities. Of note, some dentists lacked knowledge of the incubation period of 5 days (21.19%; Table 2).

**Table 2:** Respondents’ knowledge and views of COVID-19 and related infection control measures and practice provision during the pandemic period.

QUESTION	Correct response	
	No.	%
Fever, cough, and shortness of breath (days 2–14) are mild symptoms of COVID-19	3333	92.94
COVID-19 has a poor prognosis for elderly and those with comorbidities	3577	99.75
On average, COVID-19 patients show symptoms after 3–5 days after incubation	760	21.19
Dyspnoea (respiratory rate >30/min) is a major symptom of severe acute COVID-19	3331	92.89
PCR of nasal and throat swabs is a major diagnostic method of COVID-19	3306	92.19
Supportive therapy is one of the treatment options for COVID-19	3486	97.21
A COVID-19 patient can still be a disease carrier until the 14th day after symptom clearance	3170	88.40
The patients under surveillance category comprises those with a temperature >38 °C, respiratory problems, or a history of travel to a transmission area or in contact with a patient positive for COVID-19	3503	97.69
Health care workers who have had contact with COVID-19 patients without standard PPE are a high-risk group	3294	91.86
Provision of care		
Do you continue to serve patients during a pandemic?	1823	50.84
Yes	902	
a. Community health centre b. Government hospital c. Independent private practice d. Others	375	
	275	
	271	
No	1763	49.16
If you continue to practice, how many patients do you manage per day?		
Up to 10	1618	88.76
More than 10	205	11.24
Starting March 25 until now, if you continue to practice what cases are handled?		
All cases without exception	66	3.62
Emergency patients only	1139	62.48
All cases without aerosol production	618	33.90

PPE, personal protective equipment.

Regarding patient care during the pandemic, the majority—62.48%—were engaged in emergency patient care only, and surprisingly, a very small minority (3.6%; 66 respondents) treated all cases. Most who continued patient care limited the number of patients to 10 per day. A third (33.90%) of those who handled all cases did not use procedures that mitigated aerosol generation.

The use of PPE by the cohort is shown in Table 3. Most said they conducted proper precautionary procedures related to hand hygiene, PPE, and rubber dam use. In general, one-half to two-thirds of the respondents wore goggles (55.24%), face shields (69.16%), head covers (41.69%), N95 masks (52.90%), surgical masks (61.52%), total body cover gowns (61.71%), double gloves (33.85%), and boots or shoe covers (36.87%).

**Table 3:** Responses related to patient management and PPE use by the respondents (N = 1823).

Patient management	No.	%
Implement early recognition of symptoms through a screening form	1789	98.10
Implement infection prevention measures by:		
a. Hand and respiratory hygiene	1682	92.25
b. Use of appropriate PPE	1807	99.14
c. Patient gargles with povidone iodine or hydrogen peroxide	1696	93.03
d. Use of rubber dam	1243	68.18
e. Administrative reports	953	52.28
f. Carrying out environmental control	1077	59.08
PPE used:		
a. Eye protection (goggles)	1007	55.24
b. Face shield	1261	69.16
c. Head cover or hood waterproof	760	41.69
d. N95 mask or its equivalent	964	52.90
e. Surgical mask	1122	61.52
f. Gown all cover	1125	61.71
g. Apron	527	28.92
h. Double common gloves	617	33.85
i. Latex surgical gloves	448	24.57
j. Boot or shoe cover	672	36.87

PPE, personal protective equipment.

Dentists who worked in health facilities that continued to provide dental services during the pandemic were mostly those who worked in community health centres, government hospitals, and independent private practices.

The 1-way ANOVA results (supplementary material) show a comparison of knowledge, PPE usage, and patient management amongst dentists based on their workplace.

**Table 4:** Number of dentists who contracted COVID-19 during the primary and the Omicron waves of COVID-19.

Health provider	Primary waves May 2020–October 2021	Omicron wave November 2021–March 2022
Community health centres	88	165
Hospital	42	100
Private dental clinics	13	127
Government employee	10	20
Academic staff	3	24
District health office	0	6
	169	442

According to the data, there is a nonsignificant difference in dentists' knowledge across different workplaces (*F* = 1.203; *P* = .72). The range of the mean knowledge scores is 8.77 ± 1.16 to 8.92 ± 1.05.

A highly significant difference (*F* = 16.62; *P* < .001) is found in the usage of PPE amongst dentists in different workplaces. Dentists working in community health centres show lower average scores (3.33 ± 2.26), whilst those in private dental clinics exhibit higher average scores (4.36 ± 2.52). Although not statistically significant (*F* = 1.89; *P* = .057), there is a slight variation in patient management amongst groups of dentists based on their workplaces. Average patient management scores range from 6.38 ± 1.84 to 6.93 ± 1.25.

The FGDs indicated that the following factors may have contributed to the spread of COVID-19 infection amongst dental personnel in various Indonesian dental facilities: (1) the culture of officers with minimal training in pandemic-related IC (as promulgated by the IDA and the Ministry of Health) conducting surgical procedures; (2) poor design of the clinic areas to mitigate airborne infection; (3) the inability—due to financial issues—to retrofit the clinics and renovate the clinic as per pandemic IC requirements; (4) insufficiency of PPE; (5) heavy workload due to dentists working as designated COVID-19 task force officers; (6) inaccuracy and delay in receiving COVID-19 test results; and finally (7) related administrative and bureaucratic issues.

Archival data indicated that 169 Indonesian dentists contracted COVID-19 during the initial waves of the pandemic (May–October 2020; Table 4). Most of these were from community health centres (n = 88), followed by hospitals (n = 42), private dental clinics (n = 26), government employees (n = 10), and academic institutions (n = 3). The profile of those contracting COVID-19 during the third (Omicron) wave was remarkably different, with increasing numbers contracting the disease from the community health centres (n = 165), followed by hospitals (n = 100), and private dental clinics (n = 127). Unfortunately, 82 dentists died during the second wave of the COVID-19 pandemic from November 2020 to August 2021 (Delta). Further, from November 2021 to March 2022 (third wave; Omicron), this number increased to 442, although fortunately, only a single death was reported. The national records indicated that, overall, a total of 94 dentists succumbed to the disease during various phases of the pandemic.

## Discussion

Our findings indicate that during the initial waves of the pandemic, there were reports of 169 dentists contracting COVID-19, of whom 11 succumbed to the illness; the majority (52%) were from the community clinics. This finding aligns with the data indicating that the community health centres, compared to private clinics, were the health care providers that continued dental services during the pandemic. Nevertheless, it is difficult to ascertain whether those with the disease contracted the infection from the patients they treated or from the community. But a large proportion of them may have contracted COVID-19 from the dental practice, as some continued to treat patients during the period, sometimes without proper PPE due to its acute shortage during the pandemic. The early phase of the COVID-19 pandemic presented challenges for dentists in understanding the virus and its transmission. Several studies have assessed dentists' knowledge, attitudes, and practices during this period. A cross-sectional study found that most dentists had good knowledge of COVID-19, but there were gaps in understanding the incubation period, transmission, and preventive measures. Unfortunately, the questionnaire was administered during phase 1 of the COVID-19 pandemic, when dentists may have just been exposed to the information, so knowledge gaps may have been present.^20^

Regarding IC, the vast majority of surveyed dentists in Indonesia have a satisfactory understanding of COVID-19, its spread, and preventive measures (Table 1). This is not surprising, as—as in many other regions of the world—the Indonesian government was engaged in an archipelago-wide media campaign on COVID-19, its spread, and the preventive procedures directed towards both the public as well as all health care workers throughout the pandemic period and immediately thereafter.

In addition to the government campaign, the IDA also took a proactive strategy and conducted more than 15 webinars directed towards dental personnel during a 2-month period early in the pandemic. These campaigns have had a salutary effect, as most respondents in the questionnaire survey and FGD had fairly good knowledge of the guidelines and IC measures of the IDA for COVID-19 protection. Similar results have been reported from other jurisdictions such as Saudi Arabia.^21^^,^^22^

Looking at the data in some detail, when questioned on the modes of transmission, most participants believed that droplet infection followed by direct contact were the major modes of transmission. Conversely, we noted a need for more knowledge of the virology of COVID-19 in our cohort. For instance, only one-fifth (21%–26%) of the cohort gave correct responses regarding the symptomatology of COVID-19 and the viability of the virus outside of the host. This finding may be due to the reported varying survival times associated with different surfaces.^21^ This is in contrast to a majority of dentists in Pakistan providing correct responses on the infectious nature of viruses and the incubation period and symptoms.^20^ More than three-quarters of the Pakistani respondents knew the correct incubation period, as opposed to one-fifth of Indonesian dentists.^23^

Regarding continuing dental practice during the pandemic, we were surprised to note that 66 dentists treated patients, although some limited the management to only 10 patients per day. Such practices, despite the guidelines of the IDA, could be due to this very small cohort's economic and financial circumstances. However, emergency dental treatment is important in both pandemic and nonpandemic situations. The authorities should learn from this experience and set up a framework for such facilities in the event of future epidemics or pandemics.^24^

As noted in our study, there should also be simultaneous provision for distributing PPE to the designated primary care facilities when there are initial shortages and distribution challenges. The authorities should realise that both the government and nongovernmental organisations must take into consideration the initial high demand for these during pandemics and appropriate intervention measures should be taken for future events.

Dentists must follow procedures that reduce aerosol generation during pandemic periods, and our data indicate that most knew and heeded the advice. A recent Indonesian study has also shown that many dentists are aware of the current guidelines issued by the CDC and WHO for cross-infection control in dental practice, including a reduction in aerosol generation and the importance of enquiring about patient travel history and recording patient body temperature.^25^^,^^26^

As regards dentists who contracted the disease, more dentists were exposed to and died from COVID-19 in the second wave of COVID-19 in Indonesia than in the first wave. Thus, 82 dentists died in the second wave (Delta, pre-Omicron) of the COVID-19 pandemic from November 2020 to August 2021, a tremendous loss for the country's dental profession. This outcome was surprising as the dental health services were, at the time, using the protocols and guidelines that the IDA published.

A detailed review of the reasons for such a large casualty needs to be carried out and corrective measures taken for the future. In a recent report by the Indonesian Ministry of Health, it was noted that the public health service had a number of obstacles that mitigated the implementation of standard guidelines issued by the IDA. These were mainly related to the budgeting and resource issues in renovating dentists’ practices. The consensus was that the Ministry of Health would issue a technical manual/guideline for dental health services. This manual is a reinforcement of the guidebook that the IDA has issued.

The FGDs elucidated some features of IC in general and pandemic situations specifically that were needed in Indonesia. These findings are congruent with similar recent Indonesian studies.^3^^,^^13^ For instance, inappropriate design of the clinical practice, lack of financial assistance for retrofitting or renovation of infrastructure, and insufficiency of PPE were elements reiterated in these discussions. In addition, work overload in the form of dentists being assigned duties as COVID-19 task force officers and the delay in COVID-19 test results were other elements featured in FGDs as possible reasons for dental staff contracting COVID-19.

Apart from these factors, other administrative issues created IC bottlenecks. One example is the IDA's weak enforcement of IC implementation guidelines, which have been emphasised by the Ministry of Health. Thus, these organisations need to resolve these issues in the future to face new emergency IC issues during pandemics and epidemics.

## Conclusions

Taken together, our findings suggest that most dental care professionals in Indonesia had adequate knowledge and awareness of COVID-19 and IC guidelines. However, it is necessary to conduct regular media campaigns throughout the archipelago about IC procedures for all health care workers. Many dentists in the country may have succumbed to the disease due to poor IC measures or not heeding the call of the IDA to restrict practice during the pandemic. Mandatory continued professional development programmes, including lectures and workshops for all health care professionals, are required to mitigate the morbidity and mortality related to current and future epidemics or pandemics.

## Author contributions

Iwan Dewanto: conceptualisation, methodology, data curation, writing–original draft, writing–review and editing, and funding acquisition. Rosa Amalia: validation, formal analysis, visualisation, and supervision. Armelia Sari: investigation and conceptualisation. Freddy Rerdiansyah: resource data of Indonesia Dental Associatian and project administration.

## Conflict of interest

None disclosed.

## Appendix Supplementary materials

Image, application 1
