# Supplementary material for: The Response of the Indonesian Dental Community to the COVID-19 Pandemic
Source: Int Dent J. 2024 Feb 16;74(4):730–5. doi: 10.1016/j.identj.2024.01.004 (PMC11287143; doi:10.1016/j.identj.2024.01.004)
Supplement: Supplementary file 1 [file mmc1.pdf]

Supplementary Material : The results of the One-way ANOVA analysis examining knowledge, completeness of PPE usage, and patient management based on the workplace of dentists.

	Mean score (sd)	F	Sig
**Provider Knowledge**			
Community health center	8,80 (1.20)	1.203	0.72
Private Dental Clinics	8,77 (1.16)		
Primary care Clinics	8,89 (1.21)		
Private hospital	8,79 (1.16)		
Public hospital	8,92 (1.05)		
Secondary care Clinic	8,85 (1.17)		
Other (government employee)	8,81 (1.30)		
**Personal Protective Equipment (PPE)**			
Community health center	3.33 (2,26)	16.62	<0.001
Private Dental Clinics	4.36 (2,52)		
Primary care Clinics	3.15 (2.51)		
Private hospital	3.66 (2.89)		
Public hospital	4.20 (2.56)		
Secondary care Clinic	3.08 (2.82)		
Other (government employee)	2.62 (2.80)		
**Patient Management**			
Community health center	6.46 (1.50)	1.89	0.057
Private Dental Clinics	6.56 (1.44)		
Primary care Clinics	6.54 (1.45)		
Private hospital	6.93 (1.25)		
Public hospital	6.62 (1.40)		
Secondary care Clinic	6.55 (1.45)		
Other (government employee)	6.38 (1.84)		
